# Presence of diabetic retinopathy is lower in type 2 diabetic patients with non-alcoholic fatty liver disease

**DOI:** 10.1097/MD.0000000000015362

**Published:** 2019-05-03

**Authors:** Meng Zhang, Li Li, Jing Chen, Bei Li, Yutao Zhan, Chuan Zhang

**Affiliations:** a Department of Gastroenterology, Beijing Tongren Hospital, Capital Medical University, Beijing; b Department of Gastroenterology, Xian XD Group Hospital, Xi’an, Shaanxi Province, China.

**Keywords:** diabetes, diabetic retinopathy, non-alcoholic fatty liver disease

## Abstract

To analyze the association between non-alcoholic fatty liver disease (NAFLD) and the presence of diabetic retinopathy (DR) in patients with type 2 diabetes mellitus (T2DM).

Total 411 T2DM patients were divided into NAFLD and control groups. NAFLD was diagnosed by ultrasound. Retinopathy was diagnosed by fundus photography. All patients were screened based on medical history, physical examinations, and laboratory measurements.

The prevalence of NAFLD and DR in T2DM patients was 60.8% and 40.9%, respectively. The presence of DR was associated with diabetes duration, systolic blood pressure (SBP), glycated hemoglobin (HbA1c), and proteinuria (all *P* < .001) using univariate and multivariate regression analyses. The prevalence of DR was lower in patients with NAFLD than those without NAFLD (37.2% vs 46.6%, *P* = .065), and significantly lower in patients with moderate and severe NAFLD (30.2% vs 46.6%, *P* = .012; 14.3% vs 46.6%, *P* = .024). The presence of DR in NAFLD patients was associated with diabetes duration (*P* = .032) in Chi-squared analysis.

NAFLD and DR were highly prevalent in T2DM patients. Diabetes duration, SBP, HbA1c, and proteinuria were risk factors for DR in T2DM patients. The presence of DR was lower in T2DM patients with NAFLD, which was mainly due to their shorter diabetes duration.

## 1 Introduction

Non-alcoholic fatty liver disease (NAFLD) refers to the condition of fat accumulation in the liver unrelated to excessive alcohol consumption and any other specific causes of hepatic steatosis.^[1]^ It has a diverse histopathological spectrum ranging from simple steatosis without significant inflammation to steatohepatitis (NASH) to various stages of fibrosis, cirrhosis, and ultimately to hepatocellular carcinoma.^[2]^ NAFLD is strongly associated with obesity, diabetes, and insulin resistance (IR).^[3]^ IR can facilitate the accumulation of triglycerides in the liver and is a key factor in the pathophysiology of NAFLD.^[4,5]^ NAFLD is the most common cause for chronic liver disease in the world.^[6]^ The pooled overall global prevalence of NAFLD diagnosed by imaging was estimated to be 25.24%.^[7]^ The incidence of NAFLD is markedly increased with the increasing prevalence of obesity, diabetes mellitus, and the metabolic syndrome in general population. It is not only associated with increased liver-related morbidity and mortality, but also with increased mortality due to cardiovascular disease (CVD) and cancer.^[8]^ There is now growing evidence that NAFLD is a multisystem disease, affecting extra-hepatic organs and regulatory pathways.^[9]^ As a result, the effect of NAFLD on extra-hepatic organs has attracted more and more research interests.

NAFLD is common in individuals with type 2 diabetes (T2DM), which is present in up to 75% of patients with T2DM.^[10]^ Current data suggest that NAFLD can increase the risk of T2DM complications, especially vascular complications.^[11,12]^ Diabetic vascular complications can be divided into 2 categories: macrovascular and microvascular complications. Macrovascular complications include coronary artery disease and cerebrovascular disease,^[13,14]^ while microvascular complications include retinopathy and chronic kidney disease.^[15]^ There were more studies on the relationship between NAFLD and coronary artery disease of T2DM^[16,17]^ and it has been demonstrated that NAFLD is an important factor for the development of coronary artery disease in patients with T2DM.^[18–20]^ Moreover, some studies have investigated the relationship between NAFLD and chronic kidney disease of T2DM.^[21,22]^ Diabetic retinopathy (DR) is the most common chronic complication of diabetes and one of the main causes of acquired blindness in the world.^[23]^ The pathogenesis of DR has not yet been fully understood.^[24]^ To date, there is very little information on the association between NAFLD and DR. The present study was to explore whether NAFLD (as diagnosed by ultrasonography) is associated with an increased risk of DR in a clinical cohort of Chinese patients with T2DM.

## 2 Materials and methods

### 2.1 Study participants

The incidence ratio of DR in NAFLD group and control group is about 1:0.64. Sample size was calculated by PASS software (PASS 11, NCSS, LLC, Kaysville, Utah) (assuming alpha = 0.1, 1-beta = 0.8, input PASS software, calculate NAFLD Group N = 250, control group N = 160). A total of 800 inpatients with T2DM from Department of Endocrinology, Beijing Tongren Hospital, Capital Medical University were initially recruited to the present cohort study between December 2014 and December 2016. The Epidemiology Ethics Committee of Beijing Tongren Hospital approved this study protocol.

### 2.2 Clinical measurements and laboratory procedures

Body mass index (BMI) was calculated as weight in kilograms divided by height in meters squared. Waist circumference was measured at the level of the umbilicus. Blood pressure was assessed in triplicate with a standard mercury manometer. Information on name, sex, age, diabetic duration, daily alcohol consumption, and smoking status of participants was obtained by systematically inquisition. Individuals completed self-administered questionnaires, related to their medical and social history and medication usage. Fasting blood glucose (FBG), hemoglobin A1C (HbA1C), blood lipid, high sensitivity C reactive protein (HCRP), proteinuria, liver enzymes were determined by standard laboratory procedures (AU5800, Beckman Coulter, Inc. 250 S. Kraemer Boulevard Brea, CA).

Eight hundred inpatients were screened for NAFLD by abdominal ultrasound. The classification of NAFLD was carried out based on the severity of fatty liver by abdominal ultrasound according to the given criteria.^[25]^ Grade 1: no fatty liver; Grade 2 (mild): there was slight diffuse increase in the echogenicity of liver parenchyma or increased hepatorenal contrast with normal diaphragm and intrahepatic vessel borders; Grade 3 (moderate): there was moderate diffuse increase in the echoegenicity of liver parenchyma and increased hepatorenal contrast with slight impairment of diaphragm and intrahepatic vessel borders; Grade 4 (severe): in addition to the criteria for moderate steatosis, there was no visualization of posterior portion of the right lobe of liver, intrahepatic vessel borders, and diaphragm. Moreover, all patients underwent a fundus photography that was used to diagnose diabetic retinopathy according to the guidelines for clinical treatment of DR.^[26]^

### 2.3 Statistical analysis data

The SPSS statistical package 21.0 (IBM, Amund City, New York) was used for database establishment and statistical analysis. Data are presented as means ± standard deviation, median (*P*25, *P*75). The normality of variables was checked by *K-S* test. Differences were assessed by the unpaired *t* test for normally distributed variables. Differences were assessed by Wilcoxon Mann–Whitney *U* test for non-normally distributed variables. Categorical variables were checked by chi-square test and Fisher exact test. Multivariate logistic regression analysis was used to analyze the factors associated with DR in T2DM patients, OR, and 95% CI were calculated. A *P*-value <.05 was considered statistically significant.

## 3 Results

### 3.1 Study participants

**Figure 1:**
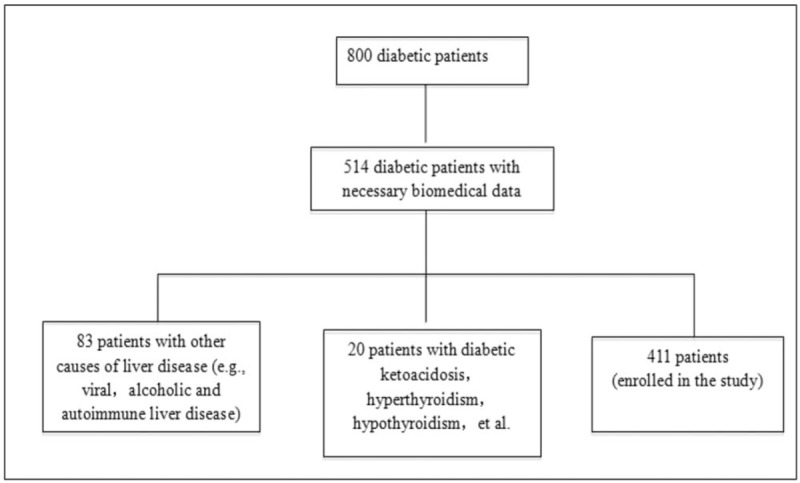
Flow chart of selection of study participants.

Total 514 of 800 patients with T2DM were screened, who had complete clinical information. Those patients included individuals who did not consume alcohol or consumed alcohol <20 g/d. Eighty-three patients with positive serology for hepatitis B or C or with a history of chronic liver disease were excluded from the study. Patients with NAFLD did not receive any medical treatment to prevent liver injury before they were recruited to this study. Twenty patients with diabetic ketoacidosis, hyperthyroidism, hypothyroidism were excluded. Total 411 enrolled patients were divided into 2 groups (NAFLD group and control group) based on the presence of NAFLD or not, diagnosed by abdominal ultrasonography to identify fatty liver disease (Fig. 1).

### 3.2 The clinical features and biochemical characteristics of participants

In the whole study population, the prevalence of NAFLD among the participants was about 60.8% (250/411). The FBG, BMI, waist circumference, waist to hip ratio, triglyceride (TG), alanine aminotransferase (ALT), aspartate aminotransferase (AST), alkaline phosphatase (ALP), and γ-glutamyl transferase (GGT) in patients with NAFLD were significantly higher than those in the control group (*P* < .05). The proportion of male patients in the NAFLD group was 42%, which was markedly lower than those in the control group (*P* < .05). There were no significant differences in age, duration of diabetes, proteinuria, systolic blood pressure (SBP), diastolic blood pressure (DBP), cholesterol, low density lipoprotein cholesterol (LDL), HbA1C, HCRP, smoking history, and family history of diabetes between 2 groups (*P* > .05) (Table 1).

**Table 1:**
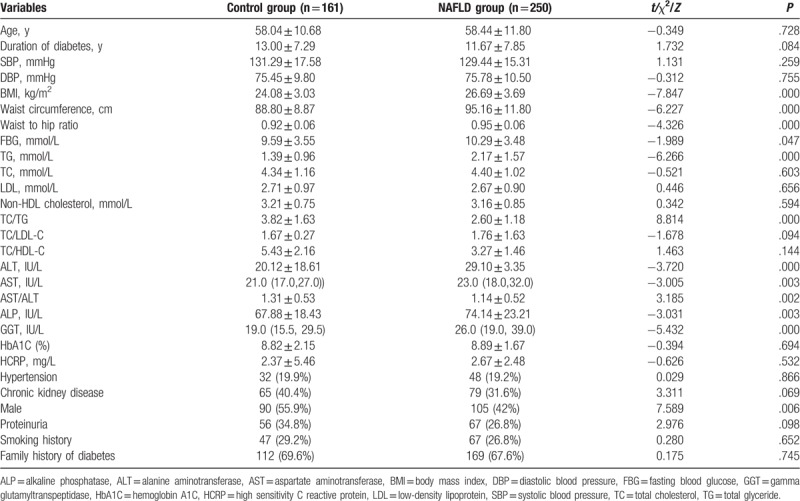
Clinical and laboratory characteristics of study subjects.

### 3.3 Prevalence of DR in participants

As shown in Table 2, the prevalence of DR in the participants was about 40.9%. The prevalence of DR was 46.6% and 37.2% in control and NAFLD groups, respectively. Compared with the control group, the prevalence of DR in the NAFLD group is lower, but the difference was not statistically significant (*P* > .05).

**Table 2:**
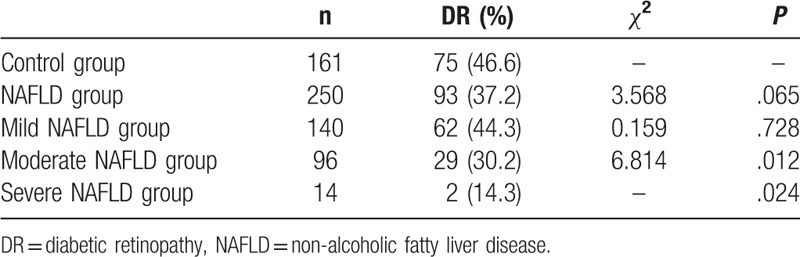
Prevalence of DR in T2DM patients with different levels of NAFLD.

According to the given criteria,^[1]^ the patients in the NAFLD group were further divided into mild NAFLD, moderate NAFLD, and severe NAFLD groups. Compared with the control group, the prevalence of DR in the moderate NAFLD group and the severe NAFLD group is significantly lower (*P* < .05), the prevalence of DR in the mild NAFLD group is lower but no statistical significance (*P* > .05).

### 3.4 Multivariable logistic regression analyses of the risk factors for DR in participants

In univariate logistic regression analysis (Table 3), HbA1c, diabetes duration, proteinuria, and SBP were significantly correlated (*P* < .05) with increased rate of retinopathy. The correlation remained significant after adjustment for age, sex, and BMI (Table 3; model 1). Moreover, after adjustment for comorbidities including CVD and chronic impaired renal function, the correlation was still significant (*P* < .05) (Table 3; model 2, 3). However, there were no statistical significance in the relationship between NAFLD and age, smoking behavior, family history of diabetes, BMI, blood lipid level, lipoproteins, and DR, which were not shown in the Table 3.

**Table 3:**
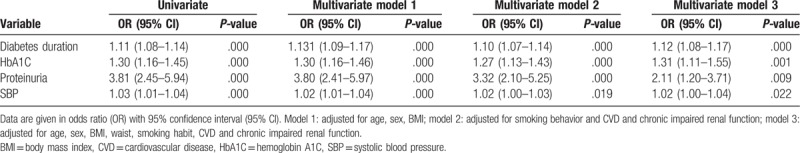
Univariate and multivariate logistic regression analyses of DR in participants.

### 3.5 The differences of the DR risk factors in patients with different degree of fatty liver

To examine the risk factors of DR in patients with different levels of fatty liver, we further graded NAFLD according to ultrasound findings. As shown in Table 4, individual diabetes duration was significantly shorter in the NAFLD group (*P* < .05). We also found that more severe fatty liver is, the shorter duration of diabetes is. However, the other risk factors of DR such as HbA1C, proteinuria, and SBP remained not statistically significant in patients with different levels of fatty liver.

**Table 4:**
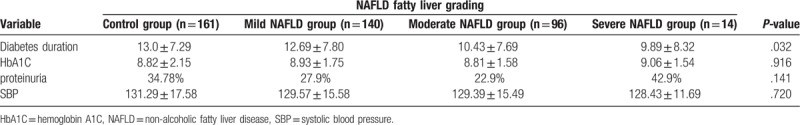
The differences of the DR risk factors in T2DM patients with different degrees of fatty liver.

## 4 Discussion

Diabetes is a common metabolic disease with a rising global prevalence. It is estimated to affect 415 million people worldwide, which accounts for almost 10% of the global adult population.^[26]^ Moreover, recent data predict that diabetes may further rise to almost 600 million worldwide by 2035.^[27]^ T2DM represents about 90% of diabetes.^[28]^ T2DM is mostly accompanied by NAFLD. The present study found that the prevalence of NAFLD in T2DM patients was 60.8%, which is similar to the 59.67% of pooled prevalence of NAFLD in T2DM patients in a meta-analysis study on 24 studies involving 35,599 T2DM patients.^[29]^ It was well-known that the relationship between T2DM and DR is complicated because the incidence of DR in patients with T2DM varied in different studies. One study from Spain showed that the DR prevalence in 14,266 T2DM patients was 14.9%.^[30]^ Another study from United Kingdom found that the prevalence of DR was 20.2% in 1062 patients with newly diagnosed T2DM. However, our result suggested that the prevalence of DR in T2DM patients was 40.9%. This result is consistent with the 40.5% of a study from China.^[31]^

As the global prevalence of diabetes increases, the number of patients with DR^[32]^ has been estimated to reach 191.0 million by 2030^[33]^ and diabetes will become the leading cause of vision loss and blindness in working-age adults.^[26]^ The identification of risk factors associated with DR development is essential for developing preventive strategies. NAFLD usually coexists with T2DM and is a confirmed risk factor for the development of CVDs in patients with T2DM.^[11]^ As a result, it is speculated that NAFLD may be a risk factor for DR in patients with T2DM. The association between NAFLD and DR in type 2 diabetes has not been studied thoroughly. Three previous studies investigated the association between NAFLD and DR in type 2 diabetes, but they presented different results. One study by Targher et al^[34]^ showed that NAFLD is associated with increased prevalence of retinopathy and is independently associated with an increased prevalence of proliferative/laser-treated retinopathy in Italian patients with type 2 diabetes. However, another study from Korean by Kim et al^[35]^ showed that NAFLD is inversely associated with the prevalence of DR in Korean patients with type 2 diabetes and one study by Lv et al^[36]^ reported that NAFLD was also negatively correlated with the prevalence of DR in Chinese patients with type 2 diabetes. Our results showed that the prevalence of DR in patients with NAFLD is slightly lower than that of DR in patients without NAFLD (*P* > .05) but the prevalence of DR in patients with moderate and severe NAFLD was significantly lower than that of DR in patients without NAFLD (*P* < .05).

Risk factors for developing any DR have been described in many studies. Sasongko et al^[37]^ found that the duration of diabetes, fasting glucose level, and hypertension are independently associated with the presence of DR in Indonesians with type 2 diabetes. Ting et al^[33]^ found that hyperglycaemia, hypertension, hyperlipidemia, and obesity are the modifiable risk factors, while the duration of diabetes, puberty, and pregnancy are the non-modifiable risk factors for DR development and progression. In a prospective cohort study, Yun et al^[38]^ demonstrated that glycemic control, diabetes duration, age, and albuminuria are important risk factors for DR development, but there is no significant relationship between DR and traditional serum lipid levels, the presence of hypertension, BMI. In a retrospective cross-sectional study, Yan and Ma^[39]^ found that fasting serum glucose concentration, HbA1c level, diabetes duration, and insulin treatment are potential risk factors for DR in northern Chinese patients with T2DM. In a prospective longitudinal follow-up study, Abougalambou et al^[40]^ found that there is a significant association between DR and diabetes duration, the presence of neuropathy, total cholesterol, and createnine clearance, but there is no significant difference between DR and age, sex, fasting plasma glucose, HbA1c, systolic BP, or diastolic BP. In the present study, we revealed that HbA1c, diabetes duration, proteinuria, SBP are risk factors for DR development, but age, smoking, family history of diabetes, BMI, and blood lipid are not associated with DR development.

We found that the incidence of DR is lower in T2DM patients with NAFLD, especially with moderate or severe NAFLD compared with that in T2DM patients without NAFLD. This result is not consistent to that NAFLD is an independent risk factor for CVD development in T2DM patients. We further analyzed those risk factors of DR in patients with different levels of NAFLD and observed that only diabetes duration is significantly associated with different levels of NAFLD. It has been demonstrated that the duration of diabetes is one of independent and the most consistent risk factors for DR.^[41]^ The occurrence of DR increases with the duration of diabetes and an 8% increase in patients with DR in each additional year was observed with progression of diabetes.^[40]^ As a result, shorter diabetes duration in patients with NAFLD may be the most important factor for lower incidence rate of DR.

### 4.1 Limitations

This study has several limitations. First, this is a cross-sectional study and cannot determine causal relationship between NAFLD and DR in patients with T2DM. Second, participants in our study were diabetic outpatients from single hospital, those results might not be generalizable to all T2DM patients. Third, the diagnosis of NAFLD and its degree was based on ultrasound imaging. The patients did not receive liver biopsy, which is the gold standard for the diagnosis and determination the degree of NAFLD. Despite these limitations, many results in present study are consistent with those reported previously by other authors. Thus, our results are valid and reliable.

### 4.2 Future directions

Prospective multi-community biopsy—proved NAFLD studies are required to elucidate the association between NAFLD and DR development in patients with T2DM. Especially the relationship between biopsy—confirmed NASH (the severe form of NAFLD) and DR in patients with T2DM is an important research direction in the future.

## 5 Conclusion

Our study demonstrated a higher incidence of DR with 40.9% in hospitalized Chinese patients with T2DM. Longer duration of diabetes, proteinuria, increased HbA1c, and SBP are risk factors for the development of DR. The presence of DR was lower in T2DM patients with NAFLD, especially patients with moderate and severe NAFLD compared with T2DM patients without NAFLD. Shorter diabetes duration may be the main cause of low rate of DR in T2DM patients with NAFLD.

## Author contributions

Yutao Zhan contributed to the conception of the study; Meng Zhang and Li Li performed the data analyses and wrote the manuscript; Bei Li, Wenjun Lin and Jing Chen contributed significantly to analysis and manuscript preparation; Chuan Zhang helped perform the analysis with constructive discussions.

**Conceptualization:** Chuan Zhang.

**Data curation:** Meng Zhang.

**Investigation:** Jing Chen.

**Methodology:** Li Li.

**Resources:** Yutao Zhan.

**Software:** Bei Li, Wenjun Lin.
